# Alcalase Potato Protein Hydrolysate-PPH902 Enhances Myogenic Differentiation and Enhances Skeletal Muscle Protein Synthesis under High Glucose Condition in C2C12 Cells

**DOI:** 10.3390/molecules26216577

**Published:** 2021-10-30

**Authors:** Yi-Ju Chen, Ching-Fang Chang, Jayaraman Angayarkanni, Wan-Teng Lin

**Affiliations:** 1 Department of Surgery, Taichung Veterans General Hospital, Taichung 40704, Taiwan; chenyiju5668@gmail.com; 2 Department of Animal Science and Biotechnology, Tunghai University, Taichung 40704, Taiwan; 3 Department of Food Science, College of Agriculture, Tunghai University, Taichung 40704, Taiwan; g08621006@thu.edu.tw; 4 Department of Microbial Biotechnology, Bharathiar University, Coimbatore 641046, Tamil Nadu, India; angaibiotech@buc.edu.in; 5 Department of Hospitality Management, College of Agriculture, Tunghai University, Taichung 40704, Taiwan

**Keywords:** sarcopenia, muscle atrophy, protein synthesis, mitochondrial biogenesis

## Abstract

Sarcopenia is an aging associated disorder involving skeletal muscle atrophy and a reduction in muscle strength, and there are no pharmaceutical interventions available thus far. Moreover, conditions such as hyperglycaemia are known to further intensify muscle degradation. Therefore, novel strategies to attenuate skeletal muscle loss are essential to enhance muscle function and thereby improve the quality of life in diabetic individuals. In this study, we have investigated the efficiency of a potato peptide hydrolysate PPH902 for its cytoprotective effects in skeletal muscle cells. PPH902 treatment in C2C12 cells showed the dose-dependent activation of the Akt/mTOR signalling pathway that is involved in skeletal myogenesis. According to Western blotting analysis, PPH902 induced the phosphorylation of Akt, mTOR proteins and induced the myogenic differentiation of C2C12 myoblasts in a differentiation medium. The phosphorylation myogenic transcription factor Foxo3A was also found to be increased in the cells treated with PPH902. In addition, treatment with PPH902 ameliorated the high glucose induced reduction in cell viability in a dose-dependent manner. Moreover, the number of myotubes in a differentiation medium reduced upon high glucose challenge, but treatment with PPH902 increased the number of differentiated myotubes. Further, the phosphorylations of AMPK and mitochondrial-related transcription factors such as PGC-1α were suppressed upon high glucose challenge but PPH902 treatment restored the protein levels. We demonstrate, for the first time, that a specific potato peptide has a therapeutic effect against sarcopenia. In addition, PPH902 improved the myogenic differentiation and their mitochondrial biogenesis and further improved myogenic protein and inhibited muscle protein degradation in C2C12 cells challenged under a high glucose condition.

## 1. Introduction

Aging is inevitably associated with a progressive decline in bodily function and composition with a decline in muscle strength and mass, a phenomenon known as sarcopenia. The consequence of this effect includes a reduction in aerobic capacity, causing reduced mobility and quality of life in elderly subjects. The annual cost, in the United States, of the health effects of sarcopenia accounts to USD 18.5 billion and the scenario is likely to be global as the aging population grows in most developed countries [1]. In spite of a huge allocation of resources, there has been no drug therapy identified for this condition [2]. The present treatment strategy recommends protein nutritional uptake and physical exercise to maintain muscle function [3,4].

Akt inhibits protein degradation by repressing the forkhead box protein (FoxO) family, leading to the expression of atrogin-1/Muscle Atrophy F-box (MAFbx) and Muscle RING-Finger Protein-1 (MuRF1) [5]. Akt stimulates protein synthesis by regulating glycogen synthase kinase3β (GSK3β).

The accumulating evidence suggests that the intake of nutraceutical foods with cholesterol-lowering functions could help the elderly to achieve health relatively easily [6]. In fact, these potentially nutraceutical foods include proteins, protein hydrolysates and peptides sourced from plants, such as soybean [7]. Soy peptides have been reported to decrease the risk of cardiovascular disease [8,9]. Alcalase was reported to hydrolyse soy protein into antioxidative hydrolysates [10]. Not only soy peptides but also potato protein hydrolysates (PPH) have been found to have antioxidative activity and exert protective effects against ethanol-induced gastric mucosal damage [11]. Potato is a major source of dietary proteins, minerals and antioxidants [12].

PPH902 is a 2-hour alcalase hydrolysed potato protein containing 9% of 200 ppm hydrolysates and possessing lipolysis-stimulating activity with efficient anti-obesity potential. PPH902 was found to be effective at reducing diabetic damage to the heart and liver by attenuating cell apoptosis and activating mitochondrial biogenesis by restoring the SIRT1 expression that was curtailed by a high-fat diet [13,14]. Nevertheless, the effect of PPH902 on skeletal muscle protection has not yet been determined. In this study, the effects of PPH902 supplementation on the skeletal muscle atrophy and high-glucose-induced myogenic aversions are investigated

## 2. Results

### 2.1. Effect of PPH902 on Cell Viability

**Figure 1:**
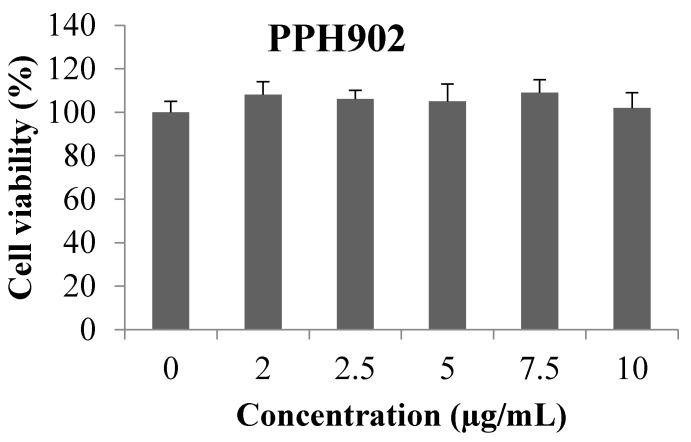
Effect of PPH902 on cell viability of C2C12 cells. Treatment of different concentration of PPH902 in C2C12 did not cause any reduction in their viability.

In order to determine the effect of PPH902 on the viability of C2C12 cells, MTT was performed, and the results showed no toxic effect of PPH902 on C2C12 cells even up to 10 μg/mL (Figure 1).

### 2.2. Effect of PPH902 on Protein Synthesis Mechanism

**Figure 2:**
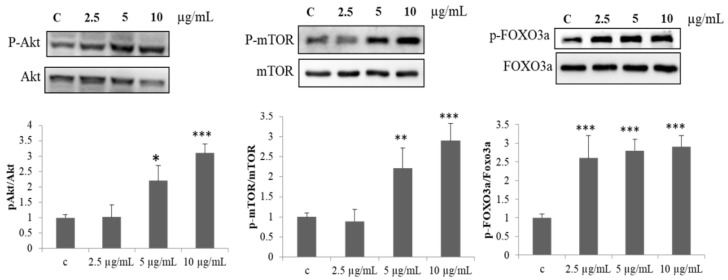
PPH902 enhances protein synthesis mechanism. Representative Western blotting shows phosphorylation of Akt and FOXO3A in C2C12 cells in response to PPH902 administration. *** *p* < 0.001, ** *p* < 0.01 and * *p* < 0.05 indicate significant differences with respect to control group.

In addition, the activation of mTOR by Akt is an important event in the skeletal myogenesis. To evaluate the potential to induce myogenic differentiation, the effect of PPH902 in activating Akt/mTOR signalling was assessed. The administration of 2.5, 5 and 10 μg/mL of PPH902 increased the phosphorylation and activation of kinases ERK, Akt and mTOR that are associated with muscle protein synthesis and elevated the regulatory phosphorylation of pro-apoptotic and matrix transcription factor FOXO3A (Figure 2).

### 2.3. Effect of PPH902 on C2C12 Cell Proliferation

**Figure 3:**
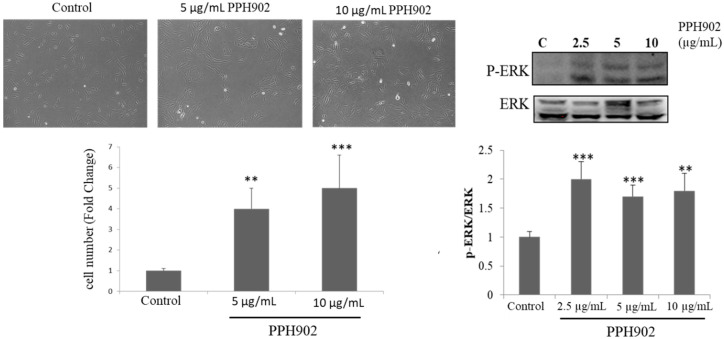
Effect of PPH902 on cell proliferation. Representative Western blotting shows phosphorylation levels of ERK. Treatment with PPH902 showed an increase in the C2C12 cell number. *** *p* < 0.001 and ** *p* < 0.01 indicate significant differences with respect to control group.

Microscopically observing cultured C2C12 cells showed difference in their proliferation rate upon treatment with different concentrations (5 and 10 μg/mL) of PPH902. C2C12 cells cultured for 24 h followed by treatment with 10 μg/mL of PPH902 for 12 h displayed an increase in the cell number and showed mesenchymal morphology. The ERK signalling pathway is involved in the myoblast proliferation. Therefore, the PPH902-induced modulations in ERK activation were accessed to evaluate its effects on myoblast proliferation. The increase in cell number upon treatment with 2.5, 5 and 10 μg/mL of PPH902 was correlated with an effective increase in ERK activation, as seen from the levels of pERK in C2C12 cells (Figure 3).

### 2.4. Effect of PPH902 on Myogenic Differentiation

**Figure 4:**
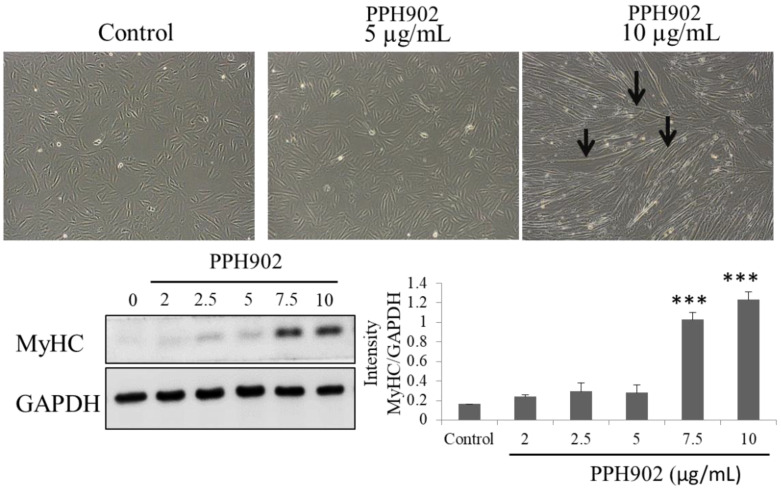
Effect of PPH902 on myogenic differentiation. Treatment with PPH902 showed a dose-dependent increase in the myogenic differentiation of C2C12 cells. Representative Western blots show an increase in the levels of MyHC in C2C12 cells upon treatment with different doses of PPH902. *** *p* < 0.001 indicates significant differences with respect to control group.

In order to understand the effects of PPH902 on muscle regeneration, their potency in inducing myogenic differentiation was examined. DMEM containing 2% horse serum induced the differentiation of C2C12 cells that are usually a flat-spindle shape to long myotubes and with thick spindle morphology within 5 days. Microscopically observing a C2C12 cell cultured in differentiation media shows that treatment with different concentrations (5 and 10 μg/mL) of PPH902 enhances myogenic differentiation (Figure 4). The myotubes in the 10 μg/mL PPH902-treated cells were clearly visible with few Y-shaped myotubes (Figure 4). In addition, analysis of the expression levels of MyHC, a muscle specific marker, indicated C2C12 differentiation. The results show a dose-dependent increase in MyHC in 5 days of PPH902 administration (Figure 4).

### 2.5. Effect of PPH902 on High Glucose Induced C2C12 Cytotoxicity

**Figure 5:**
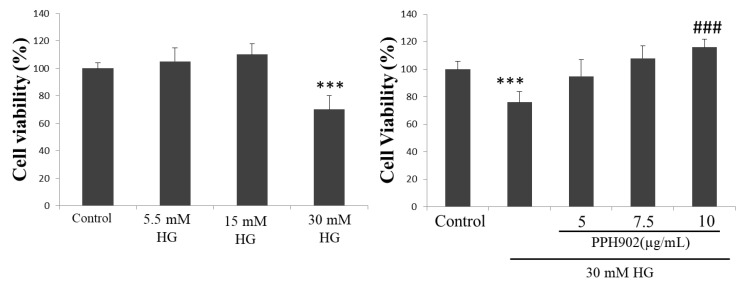
PPH902 on the effect of HG on C2C12 cells. MTT cell viability assay shows the effect of high glucose on cell viability of C2C12 cells. *** *p* < 0.001 indicates significant difference with respect to control groups and ### *p* < 0.001 indicates significance difference with respect to the high glucose challenge groups.

As determined using an MTT assay, the high glucose challenge in C2C12 cells decreased the viability of C2C12 cells in a dose-dependent manner. However, treatment with PPH902 improved the viability of C2C12 cells challenged with high glucose (HG) (Figure 5).

### 2.6. Effect of PPH902 on High Glucose Induced Myogenic Differentiation

**Figure 6:**
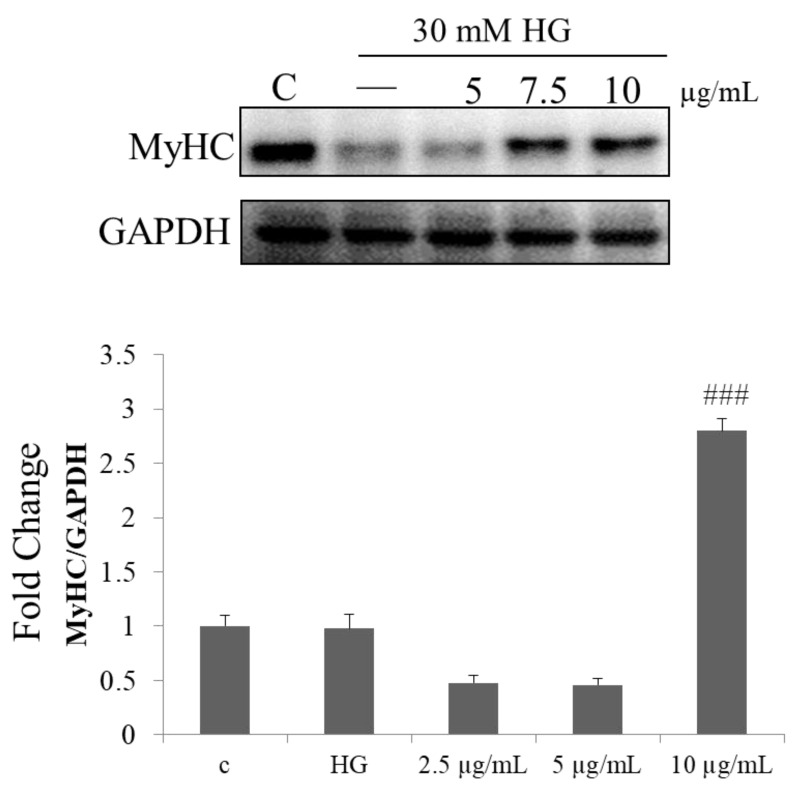
PPH902 on the effect of HG-induced myogenic differentiation. Representative Western blotting shows changes in the levels of MyHC in C2C12 cells. ### *p* < 0.001 indicates a significance difference with respect to the high glucose challenge groups.

To further confirm the effects of high glucose on myogenic differentiation and the ameliorating effect of PPH902, the expression level of the differentiation marker, MyHC, a major structural protein in myotubes was determined in C2C12 cells cultured in differentiation media. The results show a reduction in the MyHC levels upon a 30 mM high glucose challenge; however, treatment with PPH902 showed an increase in the levels of MyHC (Figure 6).

### 2.7. Effect of PPH902 on High Glucose Associated Modulations in Protein Synthesis Machinery in C2C12

**Figure 7:**
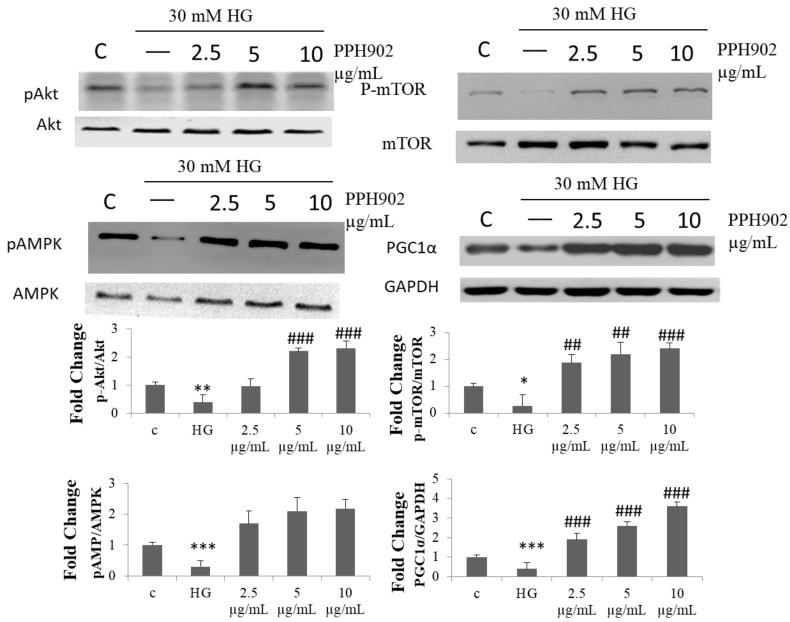
PPH902 reverses high-glucose-induced suppression of protein synthesis machinery in C2C12. Western blotting analysis shows modulations in the levels of pAkt, p-mTOR, p-AMPK and PGC1α. *** *p* < 0.001, ** *p* < 0.01 and * *p* < 0.05 indicate significant differences with respect to control groups; ### *p* < 0.001 and ## *p* < 0.01 indicate significance differences with respect to the high glucose challenge groups.

The high glucose challenge reduced the activation of mTOR and Akt kinases associated with protein synthesis and AMPK associated with mitochondria biogenesis and the associated transcription factor PGC1α (Figure 7). However, treatment with PPH902 showed a considerable increase in the active phosphorylated forms of the kinases and the expression levels of PGC1 α in C2C12 cells.

### 2.8. PPH902 Inhibits High Glucose Induced Muscle Atrophy by Downregulating Atrogin1/MAFbx and MuRF1

**Figure 8:**
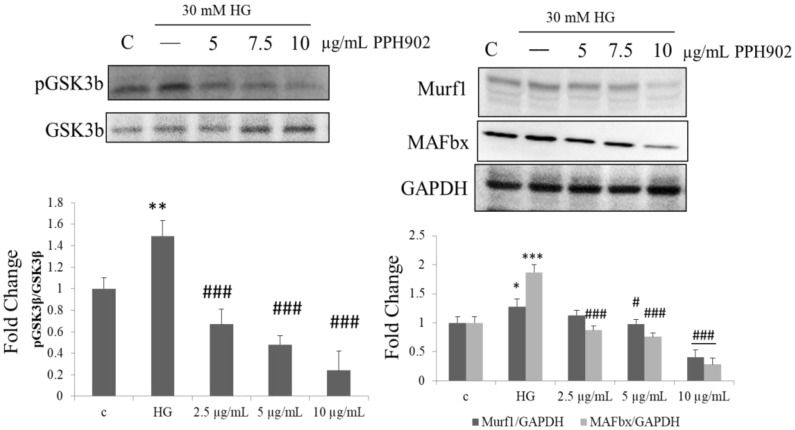
Effect of PPH902 on high-glucose-induced muscle atrophy mechanism: Representative Western blotting shows changes in the levels of GSK3b phosphorylation, MAFbx and MyHC in C2C12 cells. * *p* < 0.05, ** *p* < 0.01 and *** *p* < 0.001 indicate significant difference with respect to control groups and # *p* < 0.05 and ### *p* < 0.001 indicate significance difference with respect to the high glucose challenge groups.

Akt stimulates glycogen synthesis in the skeletal muscles via the phosphorylation and inactivation of GSK-3β, thereby inhibiting atrophy by regulating muscle-specific E3 ubiquitin ligases, namely atrogin-1 (MAFbx) and muscle RING finger 1 (MuRF1). Treatment with 30 mM of glucose showed an increase in GSK-3β with a corresponding increase in MAFbx and MuRF1. However, treatment with PPH902 suppressed the activation of GSK-3β and the expression of MAFbx and MuRF1 (Figure 8).

### 2.9. PPH902 Activates Mitochondrial Biogenesis via NRF-1 and TFAM in C2C12 Cells

**Figure 9:**
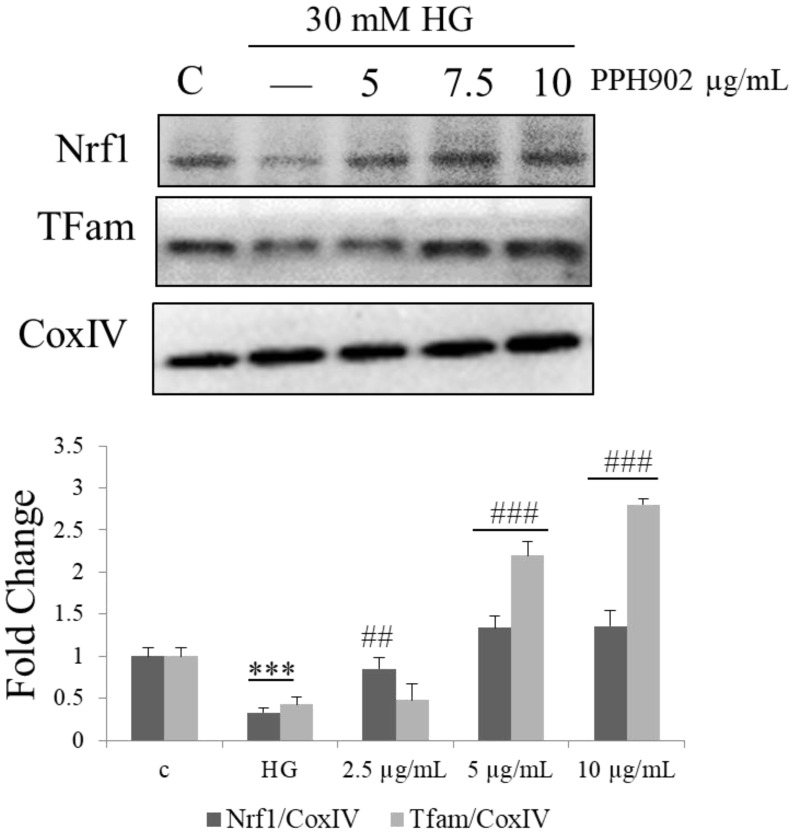
Effect of PPH902 on the activation of mitochondrial biogenesis: Representative Western blotting shows changes in the levels of NRF1 and TFAM in C2C12 cells. *** *p* < 0.001 indicates a significant difference with respect to control groups and ## *p* < 0.01 and ### *p* < 0.001 indicates a significance difference with respect to the high glucose challenge groups.

Gate keeper genes, such as PGC1 α, activate mitochondrial biogenesis in skeletal muscles by acting on their targets, such as NRF1 and TFAM. In the present study, the high glucose challenge in C2C12 cells suppressed the level of NRF-1 and TFAM and treatment with PPH902 enhanced their levels (Figure 9). Therefore, PPH902 potentially maintains mitochondrial biogenesis to enhance muscle metabolism and function.

## 3. Discussion

As the aging population increases, the concepts of quality of life and healthy aging have attained greater importance. The prominent strategy to improve the quality of life during aging is to manage the diet and improve physical exercise, thereby preserving muscle function. However, aging persons often have restrictions on their diets and their ability to perform physical exercise. In such conditions, functional food supplements can fulfil this requirement [15]. Therefore, in the present study, we have investigated the effect of PPH902 on preventing the age-associated loss of muscle function. The results show that PPH902 treatment in a culture myoblast abrogated the decline in muscle protein synthesis. PPH902 is, therefore, a potential functional peptide to improve muscle function and to reduce loss of muscle mass associated with diabetes. Further analysis shows that PPH902 administration enhanced myogenic differentiation through the upregulation of myogenic protein expression. It was also observed that PPH902 activated the Akt/mTOR pathway, which is a key cascade in skeletal muscle protein synthesis.

The present results, for the first time, demonstrate that a lipolysis potato peptide suppresses the age-associated loss of muscle cell mass. PPH902 improved the myogenic differentiation of C2C12 cells in hyperglycaemic conditions. PPH902 is known to have previously had therapeutic potential against diabetes, hypertension and obesity-associated damage to the heart and skeletal muscles [16,17]. Although it is well known that certain nutritional interventions, such as essential amino acids, milk-based proteins, creatine monohydrates, essential fatty acids and vitamin D, in combination with resistance exercise, may further enhance the beneficial effects on muscle mass and strength in aged populations [18], other natural products, such as (−)-epicatechin and epigallocatechin-3-gallate, have also been reported to favourably modulate muscle cell differentiation in aged animals [19]. However, in our study we focused on the effect of PPH902 in stressful conditions such as diabetes, which is very common when aging. Our results show that PPH902 reversed the effects of high glucose on skeletal muscle cell viability and protein synthesis. Further analysis will complete the detailed analysis of the role of PPH902 on mitochondrial biogenesis, protein degradation and protein synthesis with detailed mechanical study. In the following years, we will focus on the animal models of aging, and the complementary or substitutive effects of PPH902 on exercise will be evaluated.

## 4. Materials and Methods

### 4.1. Cell Culture

C2C12 murine myoblast cells were obtained from the Bioresource Collection and Research Center (BCRC, Taiwan). The C2C12 cells were cultured to a 70–80% confluence in growth medium containing DMEM (31600, Gibco cell culture media, ThermoFisher, Waltham, MA, USA) supplemented with 3.7 g/L sodium bicarbonate, 10% FBS, 100 U/mL penicillin and 100 μg/mL streptomycin. The cells were maintained in humidified 95% air and 5% CO_2_ at 37 °C. For a high glucose challenge, appropriate amounts of d-glucose were added to the culture media. As a treatment to the high glucose challenge, PPH902 was added to the media after 24 h of challenge and was maintained for the following 24 h under a high glucose condition. The amount of protein added to each group was normalised to 10 mg/mL with appropriate amounts of BSA dissolved in PBS. To exclude a hyperosmolar effect, an appropriate concentration of up to 30 mM mannitol was added to the control.

### 4.2. MTT Assay

To find the effects of PPH902 on cell viability, C2C12 cells cultured in DMEM for 24 h were treated with 2, 2.5, 5, 7.5 and 10 µg/mL PPH902 for 12 h. To find the effect of PPH902 on HG-induced cell damages, C2C12 cells were cultured in DMEM to a 70–80% confluence with growth medium containing 5.5 mM d-glucose (5.5 mM, control) and 24.5 mM d-mannitol. The high glucose challenge groups were supplemented with additional 5.5, 15 or 30 mM of d-glucose for 24 h. The media were then replaced with fresh DMEM containing same d-glucose concentrations with the presence of 5, 7.5 or 10 µg/mL PPH902 in the treatment groups and the cells were cultured for another 24 h. The media were then discarded and an MTT solution (5 mg/mL) dissolved in the growth media was added and incubated for 4 h at 37 °C. The MTT solution was removed, the formazon crystals formed were dissolved in DMSO and the absorbance was measured at 540 nm.

### 4.3. Differentiation of C2C12 Cells

For examining differentiation of C2C12 cells, the cells were approximately cultured with an initial density of 4 × 10^5^ cells/well in 10-centimeter culture plates and grown in growth medium. When the cells reached an 80–90% confluence, the growth medium was removed, and the cells were washed with Dulbecco’s phosphate-buffered saline and fed differentiation medium containing DMEM supplemented with 2% horse serum to induce differentiation. To examine the effects of PPH902 on myogenic differentiation, PPH902 was added to the media and the medium was changed every other day until day 6, and the PPH902 was replaced with each medium change.

### 4.4. Western Blotting Analysis

Western blotting was performed following methods reported previously [20,21,22]. In brief, the protein samples were resolved through SDS-PAGE (8–12%) and following electrophoresis, the samples were blotted onto PVDF membranes (Merck Millipore, CA, USA) for a stipulated time interval. Thereafter, the blots were blocked using 5% skimmed milk in TBST for 1 h at room temperature (RT) and concomitantly washed with TBST thrice. Subsequently, the membranes were incubated with specific primary antibodies (pAkt (sc-514032 Santa Cruz. Dallas, TX, USA), Akt (sc-5298, Santa Cruz), p-mTOR (#2971, cell signalling, Danvers, MA, USA), mTOR (#2983, Cell signalling), p-FOXO3a (#9466, Cell signalling), FOXO3a (#2497, Cell signalling), p-ERK (AP0974, Abclonal, Woburn, MA, United States), ERK (610408 BD), MyHC (ab50967, abcam), pAMPK (#2535, Cell signalling), AMPK (#2532, Cell signalling), PGC1α (sc-518025, Santa Cruz), pGSK3b, GSK3b (sc-9166, Santa Cruz), Murf1 (PA5-102695, ThermoFisher), MAFbx (ab168372, abcam, Waltham, MA, United States), Nrf1 (MA5-32782, ThermoFisher), Tfam (PA5-29571, ThermoFisher), CoxIV (#11967, Cell signalling), GAPDH (sc-32233, Santa Cruz)) overnight at 4 °C, washed with TBST, and finally incubated with respective secondary antibodies for 1 h in RT. Protein bands were visualised using an enhanced chemiluminescence (ECL) horseradish peroxidase (HRP) substrate (Millipore) and images were acquired using iBright FL1500 imaging system (Invitrogen, Carlsbad, CA, USA) and analysed with ImageJ software (version 1.4.3.67) (NIH, Bethesda, MD, USA), respectively [23,24,25]

### 4.5. Statistical Analysis

The results shown are the means ± SD of three independent experiments. Statistical analysis was performed using a one-way analysis of variants.

## Sample Availability

Samples of the compound is available from the authors.
